# Simple clinical indicators for early psoriatic arthritis detection

**DOI:** 10.1186/2193-1801-3-759

**Published:** 2014-12-22

**Authors:** Francesco Caso, Luisa Costa, Mariangela Atteno, Antonio Del Puente, Luca Cantarini, Ennio Lubrano, Raffaele Scarpa

**Affiliations:** Rheumatology Unit, Department of Medicine DIMED, University of Padova, Via Giustiniani 2, 35128 Padua, Italy; Rheumatology Unit, Department of Clinical Medicine and Surgery, University Federico II, via S. Pansini 5, 80131 Naples, Italy; Interdepartmental Research Center of Systemic Autoimmune and Autoinflammatory Diseases, Rheumatology Unit, Policlinico Le Scotte, University of Siena, Viale Bracci 1, 53100 Siena, Italy; Academic Rheumatology Unit, Department of Medicine and Health Sciences, University of Molise, Campobasso, Italy

**Keywords:** Early psoriatic arthritis, Psoriasis, Low-back pain, Dactylitis, Enthesitis

## Abstract

**Background:**

Diagnosis of psoriatic arthritis (PsA), in a period of 12 months from the onset of the first articular episode, permits of identifying the early form defined as “early PsA”. The recognition of the disease in this phase leads to better outcome.

The aim of this study was to identify peculiar clinical and/or laboratory findings that could be useful for the diagnosis of “early PsA”.

**Findings:**

Thirty-five patients with early onset of arthritis were observed. The following data were collected for each patient: family and personal history, physical examination, tender and swollen joint counts (TJC, SJC), tender entheseal count, presence of dactylitis and low back pain (LBP), and laboratory tests.

Among the 35 total patients, 24 showed skin and/or nail psoriasis or a family history of psoriasis.

The remaining 11 patients showed absence of concomitant or previous psoriasis and/or familiarity for psoriasis. The comparison between the two groups showed that patients with psoriasis had a significant presence of LBP, dactylitis and enthesitis than patients with psoriasis.

**Conclusions:**

The study confirms that the distinctive clinical findings of PsA is psoriasis, but also LBP, dactylitis and enthesitis have a relevant role in early identification. A low number of SJC and TJC are frequently observed in early phases of PsA than in other forms of early arthritis. These aspects could be mostly helpful when psoriasis is not detected or can follow arthritis in absence of familiar positivity, making difficult PsA diagnosis.

In conclusion, careful medical history, clinical examination and first-level laboratory investigations are useful to characterize early phases of PsA.

## Introduction

Psoriatic arthritis (PsA) is a chronic inflammatory arthropathy, belonging to the spondyloarthritic area, usually associated with skin and/or nail psoriasis or with its familiarity (Moll & Wright 1973). Other clinical sites such as the bowel, eye and cardiovascular system be variably involved (Scarpa et al. 2000; Niccoli et al. 2012; Shang et al. 2012; Costa et al. 2012).

The progress in the knowledge of new imaging techniques has changed the diagnostic approach to this condition (Soscia et al. 2012; Tan et al. 2014; Poggenborg et al. 2014; Gladman 2012), even if final diagnosis mainly relies on clinical evaluation performed by a rheumatologist. It usually refers to a characteristic presentation of joints, spine and enthesis inflammatory findings, a negative rheumatoid factor in presence of psoriasis or its familiarity (Duarte et al. 2012).

Diagnosis of PsA, in a period of 12 months from the onset of the first articular episode, permits of identifying the early form defined as “early PsA”. The recognition of the disease in this phase leads to better outcome. However, in the context of clinical practice, in early phases it is complex to rule out other inflammatory diseases, such as rheumatoid arthritis, undifferentiated arthritis and other spondyloarthritides (Gladman 2012).

In 2006, a set of classification criteria was developed by the CASPAR (ClASsification criteria for Psoriatic ARthritis) Study Group, with the aim of identifying standardized and homogeneous cohorts of PsA patients in clinical research context (Duarte et al. 2012). Although CASPAR Criteria do not represent diagnostic criteria, these have had a wide consensus, due to high specificity (98.7%) and good sensitivity (91.4%), confirmed also retrospectively (Taylor et al. 2006; Tillett et al. 2012). Furthermore, CASPAR criteria have been applied in some studies on early PsA showing good sensitivity (88.7%) and high specificity (99.1%), but data are still few (van den Berg et al. 2012; D'Angelo et al. 2009). While a distinctive clinical finding of PsA is psoriasis, dactylitis, enthesitis and inflammatory low-back pain should be present without a pathognomonic role. In Addition, no laboratory findings can be helpful in addressing PsA diagnosis. The aim of this study was to identify peculiar clinical and/or laboratory findings that could be useful for the diagnosis of “early PsA”.

## Patients and methods

In 7-month period, 35 patients (M/W: 7/28; mean age: 47.0 years, range: 17–78 years) with early onset of arthritis (within 12 weeks of onset), attending Rheumatology Unit of University Federico II of Naples, were observed. The average onset of arthritis was 5.6 months (range 2–12 months). The following data were collected for each patient: family and personal history, including familiar and/or previous psoriasis, physical examination, tender and swollen joint counts (TJC, SJC), tender entheseal count, presence of dactylitis and low back pain. Evaluation of rheumatoid factor (RF), anti-cyclic citrullinated peptide antibodies (anti-CCP) and inflammatory indices, including erythrocyte sedimentation rate (ESR) and C-reactive protein (CRP), were performed.

Written informed consent was obtained from the patient for the publication of this report.

Statistical analysis was performed using the SPSS software, version 18 (SPSS inc, Chicago, Ill). All variables were normally distributed. Analysis of variance (ANOVA) was used to assess differences between group means.

## Results

Among the 35 total patients, twenty-four showed skin and/or nail psoriasis or a family history of psoriasis (M/W: 5/19, mean age 44.9 years, range 17–73 years; mean duration of articular disease 5.8 months, range 2–12). The remaining eleven patients showed absence of concomitant or previous psoriasis and/or familiarity for psoriasis (M/W: 2/9, mean age 51.5 years, range 28–78 years; mean disease duration 5.0 months, range 2–12).

The comparison between the two groups (Table 1) showed that patients with psoriasis had a significant presence of inflammatory back pain, dactylitis and enthesitis (respectively p <0.001, p <0.001 and p <0.01), whereas patients without psoriasis showed a greater TJC (p <0.001) SJC (p <0.001), and RF positivity (p <0.01) than patients with psoriasis. In the group of patients with psoriasis, the application of CASPAR criteria led to a PsA diagnosis in all the patients, while none of the patients in the group without psoriasis was satisfying these criteria.

**Table 1:** **Clinical and laboratory findings of early arthritis patients with and without psoriasis**

	Early arthritis patients with psoriasis (n. 24)	Early arthritis patients without psoriasis (n.11)	***p***
TJC	6.0 (0–28)	13.45 (8–24)	<0.05
SJC	0.9 (0–5.0)	2.54 (0–10)	<0.05
LBP, n (%)	16; 66.6%	0	<0.001
Dactylitis, n (%)	11; 45,8%	0	<0.001
Enthesitis, n (%)	5; 20.8%	0	<0.01
ESR > 15 mm/1 h, n (%)	6; 25%	11; 100%	ns
CRP > 0.5 mg/dL, n (%)	6; 25%	11; 100%	ns
RF positivity, n (%)	0	9; 81.8%	0.01
Anti-CCP positivity, n (%)	0	4; 36.3%	ns
Uveitis, n (%)	3; 12.5%	0	ns

Data expressed as mean ± range, unless otherwise indicated.

## Conclusions

For a long time, PsA has been considered a low grade inflammatory condition and only most recently increasing data have provided evidence on the severe impact of this disease, establishing at same time the importance of an early diagnosis and treatment (Gladman 2012; Scarpa et al. 2011).

The CASPAR criteria consist of established inflammatory articular disease with at least a sum of three points from the following: current psoriasis (2 points), a history of psoriasis (1 point), a family history of psoriasis (unless current psoriasis was present or there was a history of psoriasis; 1 point), dactylitis (1 point), juxta-articular new bone formation (1 point), RF negativity (1 point), and nail dystrophy (1 point) (Taylor et al. 2006).

In this study, co-occurrence of inflammatory back pain and dactylitis were present respectively in above 46% and 66% of patients with psoriasis and were very helpful in addressing early PsA diagnosis. Enthesitis was detected in above 21% of patients with psoriasis.

Patients with psoriasis showed a lower number of TJC and SJC, than patients without psoriasis; previous episode of uveitis was referred in three of 24 patients with psoriasis and in no one of patients without psoriasis.

With regard to laboratory findings, all the patients without psoriasis showed increased ESR and C-RP, RF and anti-CCP positivity was found in above 82% and 36% of them. Differently, only 25% of patients with psoriasis showed increase of ESR and CRP and no one RF and anti-CCP positivity.

This study confirms that the distinctive clinical findings of PsA is psoriasis, but also inflammatory low-back pain, dactylitis and enthesitis have a relevant role in early identification. A low number of SJC and TJC, as well anti-CCP and RF negativity and no increase of ESR and C-RP are most frequently observed in early phases of PsA than in other forms of early arthritis. These aspects could be mostly helpful when psoriasis is not detected or can follow arthritis in absence of familiar positivity, making difficult PsA diagnosis.

The study shows several limitations: mainly the number of patients which is low and for this reason the results need to be supported by studies on larger populations.

Although the progress in the knowledge of new imaging techniques has changed the diagnostic approach to PsA (Soscia et al. 2012; Tan et al. 2014; Poggenborg et al. 2014; Gladman 2012), but PsA diagnosis still relies mainly on clinical evaluation.

Therefore, the results of the present observation may be interesting from practical point of view. The key message is that careful medical history, clinical examination and first-level laboratory investigations such as ESR, CRP, anti-CCP and FR are reasonably useful to characterize early phases of PsA. Delay in PsA diagnosis significantly contributes to poor outcome of the patient, so early identification of simple and helpful indicators could allow for the characterization of “early PsA”.

## Abbreviations

TJC: Tender joint counts
SJC: Swollen joint counts
LBP: Low-back pain
ESR: Erythrocyte sedimentation rate
CRP: C-reactive protein
RF: Rheumatoid factor
anti-CCP: anti-cyclic citrullinated peptide antibodies.
